# ZmIBH1-1 regulates plant architecture in maize

**DOI:** 10.1093/jxb/eraa052

**Published:** 2020-01-28

**Authors:** Yingying Cao, Haixia Zeng, Lixia Ku, Zhenzhen Ren, Yun Han, Huihui Su, Dandan Dou, Huafeng Liu, Yahui Dong, Fangfang Zhu, Tianyi Li, Qiannan Zhao, Yanhui Chen

**Affiliations:** 1 College of Agronomy, Synergetic Innovation Center of Henan Grain Crops and National Key Laboratory of Wheat and Maize Crop Science, Henan Agricultural University, Zhengdong New Area, Zhengzhou, Henan, China; 2 RWTH Aachen University, Germany

**Keywords:** DAP-seq, leaf angle, maize, map-based cloning, plant architecture, regulatory network, RNA-seq, *Zea mays*

## Abstract

Leaf angle (LA) is a critical agronomic trait in maize, with more upright leaves allowing higher planting density, leading to more efficient light capture and higher yields. A few genes responsible for variation in LA have been identified by map-based cloning. In this study, we cloned maize *ZmIBH1-1*, which encodes a bHLH transcription factor with both a basic binding region and a helix-loop-helix domain, and the results of qRT-PCR showed that it is a negative regulator of LA. Histological analysis indicated that changes in LA were mainly caused by differential cell wall lignification and cell elongation in the ligular region. To determine the regulatory framework of *ZmIBH1-1*, we conducted RNA-seq and DNA affinity purification (DAP)-seq analyses. The combined results revealed 59 ZmIBH1-1-modulated target genes with annotations, and they were mainly related to the cell wall, cell development, and hormones. Based on the data, we propose a regulatory model for the control of plant architecture by *ZmIBH1-1* in maize.

## Teaser

*ZmIBH1-1* encodes a bHLH transcription factor that affects the leaf angle in maize primarily through differential cell wall lignification and cell elongation in the ligular region.

## Introduction

The leaf angle (LA) in maize is formed by the leaf blade bending away from the main stem, and it has been used as an important agronomic trait for high-density planting in modern varietal breeding, as hybrids with upright leaves show a yield advantage in crop simulation models, field experiments, and breeding programs (Duncan, 1971; Pepper *et al.*, 1977; Duvick, 2005; Duvick *et al.*, 2010; Li *et al.*, 2011; Wang *et al.*, 2011). A better understanding of the molecular mechanisms underlying the determination of LA will therefore contribute to the breeding of new maize varieties with improved plant architectures.

Numerous studies have been conducted to investigate the natural variation in LA (Mickelson *et al.*, 2002; Lu *et al.*, 2007; Ku *et al.*, 2010; Tian *et al.*, 2011; Wang *et al.*, 2011) and have demonstrated that it is a quantitative trait controlled by several quantitative trait loci (QTL)/genomic regions (Moreno *et al.*, 1997; Yu *et al.*, 2007; Ku *et al.*, 2011; Dong *et al.*, 2018). Most of the QTL studies of LA in maize have been based on primary mapping (Mickelson *et al.*, 2002; Lu *et al.*, 2007, 2018; Ku *et al.*, 2010, 2012; Tian *et al.*, 2011) and only a few genes have been characterized through map-based cloning (Ku *et al.*, 2011; Zhang *et al.*, 2014). Several genes regulating LA in maize have been identified by mutant screening, most of which are related to ligule development and hormone metabolism, and are members of the YABBY gene family. Genes for a liguleless phenotype in maize affect plant architecture through an auxin signaling pathway (Muehlbauer *et al.*, 1999), and the absence of the ligular tissue results in erect leaves. *Liguleless1* and *Liguleless2* (Moreno *et al.*, 1997; Walsh *et al.*, 1998; Muehlbauer *et al.*, 1999; Moon *et al.*, 2013) are transcription factors that are necessary for the correct positioning of the blade–sheath boundary (Harper and Freeling, 1996). *Liguleless narrow* encodes a putative serine threonine kinase (Muehlbauer *et al.*, 1999) involved in ligule formation in the maize B73 background. Maize mutants deficient in brassinosteroids (BRs) have shortened internodes and twisted, dark-green, erect leaves and feminized male flowers. *Nana plant1* (*na1*) has a mutation in a gene homologous to *DET2* that encodes a 5α-reductase enzyme in the BR biosynthesis pathway (Hartwig *et al.*, 2011) and *brd1* has a mutation in the gene encoding brC-6 oxidase (Makarevitch *et al.*, 2012). Kir *et al.* (2015) used a transgenic RNAi approach to generate plants partially deficient in BRI1 which was the first report of BR signaling affecting plant development in maize. *Zmbri1*-RNAi plants have a dwarf phenotype with shortened internodes, erect leaves, and reduced auricle tissue formation, indicating a conserved function of BR signaling pathways in maize. *DROOPING LEAF* genes have also recently been reported in maize. *Drooping leaf1* (*drl1*), encodes a putative transcriptional regulator with zinc-finger and YABBY domains. This is a pleiotropic mutant characterized by reduced midribs and distally extended auricles along the medial domain of the blade–sheath boundary, and it also affects leaf length and width, LA, and internode length and diameter (Strable *et al.*, 2017). In contrast to rice, *drl*s in maize play important roles not only in the leaf but also in the stem architecture.

Thus, although some studies have identified genes that alter LA, the regulatory mechanisms and networks that control this trait still remain unclear. In a previous study we demonstrated that the major QTL associated with LA in maize, *qLA2-1*, is mapped to the short arm of chromosome 2 in the bin 2.01 region between the markers umc1165 and bnlg1297, and that it could explain 18.43% of the LA variation in the F_2_ population (Ku *et al.*, 2012). In this current study, we used a map-based approach to clone the candidate gene *ZmIBH1-1* and characterized it as a typical basic helix-loop-helix (bHLH) transcription factor that negatively regulates LA in maize. DNA affinity purification (DAP)-seq analysis and transcriptomic analysis revealed that ZmIBH1-1 directly regulates genes involved in cell wall modification, cell development, and hormone responses. This results in alterations to cell wall lignification and cell size in the ligular region of the leaf. Our findings not only reveal a new molecular function for bHLH transcription factors in the regulation of maize plant architecture, but also provides a possible gene network that regulates LA. This provides the framework for a more comprehensive study of maize plant architecture and establishes a better understanding of the function of bHLH transcription factors.

## Materials and methods

### Plant materials and growth conditions

The fine-mapping populations of maize (*Zea mays*) were generated by using Yu82 as the donor and crossing to the recurrent parent Yu87-1. Since 2008, we have generated BC_3_F_2_ (6529 plants), BC_4_F_2_ (7477 plants), BC_5_F_2_ (1837 plants), and BC_3_F_3_ (1687 plants), BC_4_F_3_ (3465 plants), and BC_5_F_3_ (6682 plants). These populations were grown at two experimental locations, namely in Zhengzhou, Henan, in the spring of 2009 and 2010, and in Sanya, Hainan, in the winter of 2008, 2009, and 2010). Phenotyping of these populations has been described previously by Ku *et al.* (2010). DNA extraction for DNA affinity purification (DAP)-seq analysis was conducted using a whole leaf (leaf 7) from Yu82 at the V7 plant growth stage (when the height of the ligule in leaf 7 is the same as that of leaf 6).

### Phenotypic analysis

Based on the results of fine-mapping, a Yu87-1 near-isogenic line (NIL) was obtained. The genetic background of this NIL was the same as Yu87-1 except for the region containing *ZmIBH1-1*, which was derived from Yu82 through foreground selection using flanking markers and background selection using 216 polymorphic SSR markers. The NIL was selected on the basis that it had a background recovery rate >95% with LA <15° (Ku *et al.*, 2010), i.e. the same as Yu82 and considerably smaller than Yu87-1. The inbred lines Yu87-1, NIL, and Yu82 were planted in six-row plots at the same density. At 10 d after pollen shedding, 30 plants from each plot were chosen randomly for evaluation of LA (using all leaves above the uppermost ear).

### Fine-mapping of *qLA2-1*

To develop molecular markers for fine-mapping, bacterial artificial chromosome (BAC) sequences of the B73 genome in the region flanked by um1165 and bnlg1297 on chromosome 2 were obtained from the Maize Genetics and Genomics Database (https://www.maizegdb.org/gbrowse). Simple sequence repeats (SSRs) were identified using the SSR Hunter software (https://en.bio-soft.net/dna/SSRHunter.html). Primers were designed using the Premier 5.0 software (Lalitha, 2000) with a PCR product size of <300 bp. Genomic DNA was extracted from fresh, immature maize leaves from all of the mapping populations. SSR markers were used initially to screen parental lines to identify polymorphic markers for linkage mapping and QTL analysis. PCR products were separated by electrophoresis on a 6% polyacrylamide gel followed by sliver staining for visualization.

### *ZmIBH1-1* cloning and phylogenetic analysis

To isolate full-length genomic DNA (gDNA) sequences for *ZmIBH1-1*, immature leaves were collected separately from Yu87-1, NIL, and Yu82. DNA extraction was performed according to the method described by Murray and Thompson (1980). The full-length gDNAs were amplified using the gIBH1-1 primers (Table S2 at Dryad Digital Repository; http://dx.doi.org/10.5061/dryad.18tk64p;Cao *et al.*, 2020). The PCR products were purified and sequenced directly.

Immature leaves were collected from Yu87-1, NIL, and Yu82 and total RNA was prepared using TRIzol^®^ reagent (Invitrogen) according to the manufacturer’s instructions. First-strand cDNA was synthesized from the total RNA and used as a template for reverse-transcriptase (RT)-PCR. The cIBH1-1 primer was designed to amplify the full-length *ZmIBH1-1* cDNA (GenBank accession no. MN161779) using one-step RT-PCR (Table S2 at Dryad). The amplified products were purified and sequenced directly. ZmIBH1-1-like protein sequences from other species were obtained using BLAST searches at the NCBI database (http://www.ncbi.nlm.nih.gov). The identified sequences were aligned using the Mega6 software (https://www.megasoftware.net/).

### CRISPR-Cas9-mediated genome editing in *Setaria*

CRISPR/Cas9-mediated genome editing was applied to *Setaria viridis* to induce targeted mutagenesis of orthologous *SvIBH1-1* to create loss-of-function alleles. gRNAs were designed using the CRISPOR online tool http://crispor.tefor.net/. *Agrobacterium*-mediated transformation of mature seed-derived callus was applied to transform the destination vectors to the ME34 background (Van Eck *et al.*, 2017).

### Protoplast transient assays

Protoplasts of tobacco (*Nicotiana tabacum*) were prepared from BY-2 suspension cells by following the method of Miao and Jiang (2007), with modifications. Tobacco plants were grown at 24 °C and subcultured every 7 d. Protoplasts were prepared from the cells 5 d after subculture. Cell walls were digested at room temperature for 2 h in a solution containing 1% (w/v) cellulase Onozuka R-10 (Serva), 0.1% (w/v) pectinase (Sigma), 0.5% (w/v) Macerozyme RS (Serva), and 0.25 M mannitol. The isolated protoplasts were transformed with 20 µg each of the reporter and effector constructs or mock DNAs using the polyethylene glycol (PEG) method.

*35S-ZmIBH1-1* contains the Cauliflower Mosaic Virus (CaMV) 35S promoter driving *ZmIBH1-1* expression, and *35S-LUC* contains the firefly luciferase driven by the constitutive CaMV-35S promoter. The reporter gene construct (UAS-GUS) and effector constructs (VP16, Gal4, and IAA17) have been described previously by Tiwari *et al.* (2001). The ZmIBH1-1-GAL4 effector construct contains the full-length *ZmIBH1-1* coding sequence fused to the N-terminus of the Gal4 DNA-binding domain under the control of the CaMV-35S promoter. The 35S-LUC construct was co-transformed as an internal control to normalize the GUS reporter gene expression. GUS and LUC enzymatic assays were performed according to Gampala *et al.* (2001).

### Subcellular localization

Full-length cDNA sequences of *ZmIBH1-1* were cloned into the vectors pSAT1-cCFP-C and pSAT1-nVenus-C (Biovector NTCC Inc., Beijing, China) to create *ZmIBH1-1-YFP*. The *ZmIBH1-1-YFP* plasmids were co-transformed into tobacco protoplasts following standard protocols. After incubation overnight in the dark at 24 °C, nuclei were stained with DAPI. eYFP and DAPI fluorescence in the protoplasts were monitored sequentially with a confocal microscope (Zeiss) using the following respective wavelengths: excitation, 514 nm and 405 nm; and detection, 527 nm and 488 nm.

### Safranin staining

Safranin staining for lignin was performed on fully expanded leaf 7 at the V7 stage. The ligular tissue was sectioned by hand to provide both cross- and longitudinal sections. Fresh sections were placed in 0.01% safranin solution for 12 min, then rinsed with 70% ETOH and ddH2O for 1 min each. The samples were mounted in paraffin oil and imaged under a light dissecting microscope.

### RNA-seq sampling

Samples of tissue were taken from the leaf base (1 cm length), the middle of the leaf blade (3 cm), and the leaf tip (10 cm) of NIL and Yu87-1 plants. In each case, the samples from 10 plants were pooled together to form one biological replicate. Three biological replicates were used for the RNA extraction. The RNA-seq library was prepared using a QuantSeq 3´ mRNA-Seq Library Prep Kit FWD for Illumina (Lexogen, Austria). Library DNA was checked for concentration and size distribution in an Agilent 2100 Bioanalyzer before sequencing with an Illumina HiSeq4000 system according to the manufacturer’s instructions.

### RNA-seq data analysis

Raw reads were adapter and quality trimmed (Q≥20) using Trim Galore (www.bioinformatics.babraham.ac.uk/projects/trim_galore/). One of the replicates each from the NIL samples for the base and mid-region of leaf 7 yielded very low read counts and hence only two replicates were used for these samples. Expression values (TPM) were generated using Salmon (Patro *et al.*, 2017) in quasi-mapping mode with the following parameters: --noLengthCorrection, --incompatPrio 0.0, and --libType SF. Maize genome annotation v3.31 was used. Gene level read counts were obtained from Salmon and used for analysis of differential gene expression by the R-based EdgeR package v3.16.5 (Robinson *et al.*, 2010). Genes with more than 5 counts per million (cpm) reads in at least three samples were kept. Estimation dispersion was performed using the glmQLFit function with robust =TRUE parameter in EdgeR (Robinson *et al.*, 2010). Significance tests were performed using glmTreat with lfc=log2(1.5). Significant genes with more than two-fold change and FDR≥0.05 were selected for further analysis.

### Real-time PCR analysis

Quantitative real-time PCR was performed using an iCycler IQ real-time PCR detection system (Bio-Rad) with SYBR Green Real-Time PCR Master Mix (ABI) according to the standard protocol. Specific primers were designed (Table 2 at Dryad), and the experiments were performed using two sets of independent RNA samples with *tubulin* as the internal control gene. All the RNA samples used were the same samples as for the RNA-seq assay. Quantifiable differences in gene expression were analysed using the 2^–∆∆*C*T^ method (Livak and Schmittgen, 2001).

### DAP-seq sampling

DAP-seq was performed by following the method described by O’Malley *et al.* (2016). First, a DAP-seq genomic DNA (gDNA) library was prepared by attaching a short DNA sequencing adaptor on to purified and fragmented gDNA. The adapter sequences were truncated Illumina TruSeq adapters; the TruSeq Universal and Index adapters corresponded to the DAP-seq Adapter A: CACGACGCTCTTCCGATCT; and Adapter B: GATCGGAAGAGCACACGTCTG. The DAP gDNA library was prepared using a kit from NEBNext^®^ DNA Library Prep Master Mix Set for Illumina^®^ (NEB, #E6040S/L). *ZmIBH1-1* was fused to the HaloTag using a kit from pFN19K HaloTag T7 SP6 Flexi Vecto (Promega #G184A). *ZmIBH1-1* fused to HaloTag was expressed using a TnT SP6 High-Yield Wheat Germ Protein Expression System (L3260, Promega), and then was purified using Magne HaloTag Beads (G7281, Promega). The Magne HaloTag Beads and *ZmIBH1-1*-HaloTag mixture were incubated with 500 ng DNA library in 40 µl PBS buffer with slow rotation in a cold room for 1.5 h. The beads were washed five times with 200 µl PBS + NP40 (0.005%), resuspended in PBS buffer, the supernatant was removed, and 25 µl EB buffer was added and incubated for 10 min at 98 °C to elute the bound DNA from the beads. The correct DAP-seq library concentration to achieve a specific read count was calculated based on library fragment size. Negative control mock DAP-seq libraries were prepared as described above without the addition of protein to the beads.

### DAP-seq data analysis

We defined target genes as those that contained DAP-seq peaks located within the transcribed regions of genes, in introns, or 5 kb upstream of the transcription start site (TSS), or 5 kb downstream of the transcription termination site (TTS). DAP-seq reads were aligned to the maize genome using Bowtie 2 (Langmead and Salzberg, 2012), which supports gapped and paired-end alignment modes. We ran Bowtie version 2.2.3 with default parameters and reported only unique alignments. DAP-seq peaks were detected by MACS2 (Zhang *et al.*, 2008). We used MACS version 2.0.10 with default parameters, as duplicates were allowed, and the q-value <0.05. Core motifs were identified by MEME-ChIP (Machanick and Bailey, 2011).

### 3´-UTR transcription level test assay

To verify the association between the extracted single-nucleotide polymorphisms (SNPs)/indels and LA, the 3´-untranslated regions (UTRs) of *ZmIBH1-1* were amplified from Yu82 and Yu87-1 and cloned into the pGreenII0800-LUC vector. Transient dual-luciferase assays were performed in *N. benthamiana* leaves. After infiltration, plants were kept at room temperature with a 14/10-h light/dark regime. Leaf protein was extracted 48 h later using a passive lysis buffer (E1910, Promega). The LUC activity was measured using a GloMax®20/20 Luminometer (Promega). Then, 100 µl of Stop and Glow Buffer was added to the reaction and *Renilla* luciferase (REN) activity was measured. The ratios between LUC and REN activities were measured three times.

### Electrophoretic mobility shift assays

^32^P-labeled DNA fragments containing specific DNA sites were incubated with the ZmIBH1-1 protein, and electrophoretic mobility shift assays (EMSAs) were performed according to Carey *et al.* (2013).

## Results

### Fine-mapping of *qLA2-1*

**Fig. 1.**
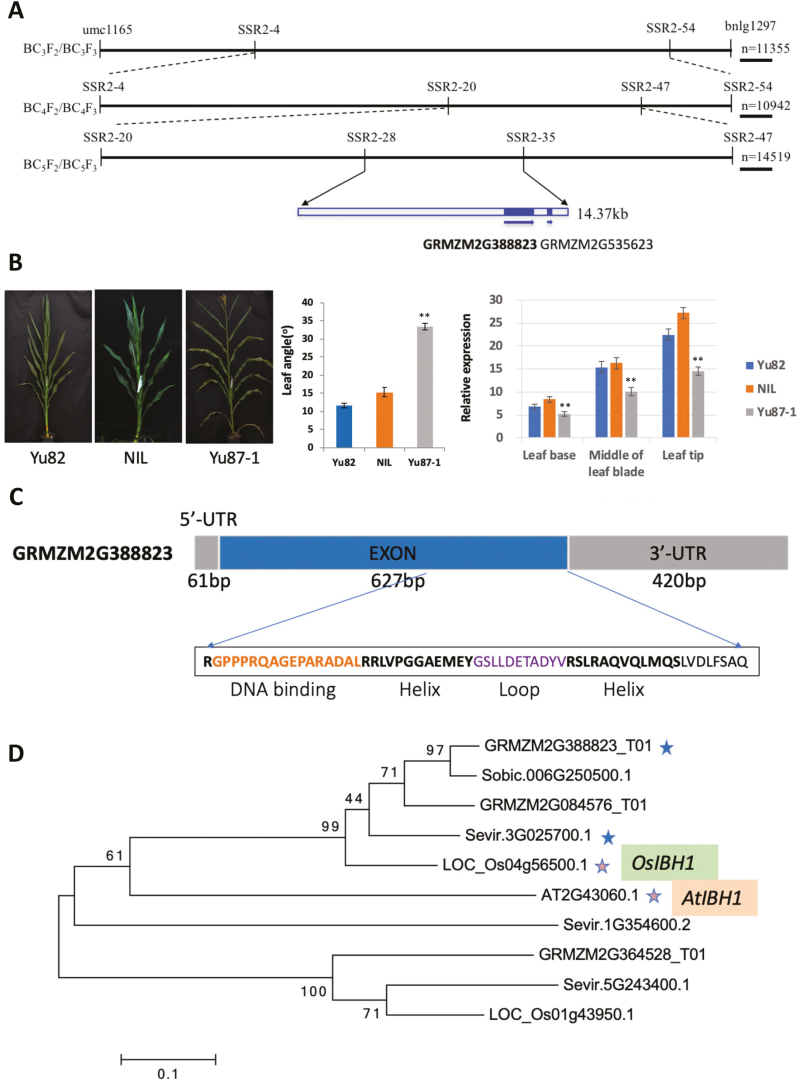
Fine-mapping of maize *ZmIBH1-1*. (A) Summary of the fine-mapping. Recombinant populations are shown on the left, the molecular markers are labeled, and the shaded boxes represent exons in the genes. The arrows indicate the direction of the genes. (B) The images show the plant architecture and leaf angles of the parental lines Yu82 and Yu87-1, and the near-isogenic line (NIL), which has the same genetic background as Yu87-1 except for the region containing *ZmIBH1-1*, which is derived from Yu82. The graphs show mean values (±SD) of leaf angle for all the leaves above the upper ear measured in 30 plants at 10 d after pollen shedding, and the relative expression levels of *ZmIBH1-1* in three regions of leaf 7, sampled at growth stage V7. Data are means (±SD) of *n*=10 replicates and are relative to expression of *tubulin*. Significant differences between the NIL and Yu87-1 were determined using Student’s *t*-test: **P<0.01. (C) Structure and sequence and of the ZmIBH1-1 protein. The sequence of the bHLH domain is shown. (D) Phylogenic analysis of IBH1 in rice (*Oryza sativa*, Os), sorghum (*Sorghum bicolor*, Sobic), Arabidopsis (AT), *Setaria virdis* (Sevir), and in maize.

*qLA2-1* in the 2.02 region has previously been identified as a major QTL that contributes 18.43% of phenotypic variation in leaf angle (LA) (Ku *et al.*, 2012; Table S1 at Dryad). For fine-mapping, the Yu82 (donor) and Yu87-1 (receptor) parental inbred lines were used to create NILs of Yu87-1 (Yu87-1_NIL). The LA of Yu87-1 was 35° whilst that of Yu87-1_NIL was 18.24° greater, confirming that *qLA2-1* contains an important locus controlling LA in maize (Fig. 1B).

In order to fine-map the *qLA2-1* locus, multiple backcross and self-pollinated mapping populations were developed (see Methods). A total of 60 newly developed polymorphic SSR markers (Table S2 at Dryad) located in the *qLA2-1* interval were used to identify recombinant genotypes for development of near-isogenic lines (NILs). The *qLA2-1* locus was located to a 14.37-kb region between the markers SSR2-33 and SSR2-34 (Fig. 1A).

Scans of this 14.37-kb interval of the maize B73_V3 reference genome identified two candidate genes in this region, *GRMZM2G388823* and *GRMZM2G535623*. Characterization of gene expression was performed by examining published RNA-seq datasets (Wang *et al.*, 2014*b*) and by qRT-PCR analysis. The results suggested that *GRMZM2G535623* was not expressed in developing leaf tissues, while *GRMZM2G388823* was expressed in leaf tissues at relatively high levels, with the highest expression near the leaf tip (Fig. 1B).

*GRMZM2G388823* encodes a 208-amino bHLH transcription factor that has both a basic binding region and a helix-loop-helix domain (Fig. 1C). Protein BLAST searches and phylogenic analysis showed that *GRMZM2G388823* is one of the two homologous genes of rice *OsIBH1* and Arabidopsis *AtIBH1* (Fig. 1D), and therefore we named it as *ZmIBH1-1*.

### Functional verification using CRISPR-Cas9-mediated genome editing in *Setaria*

**Fig. 2.**
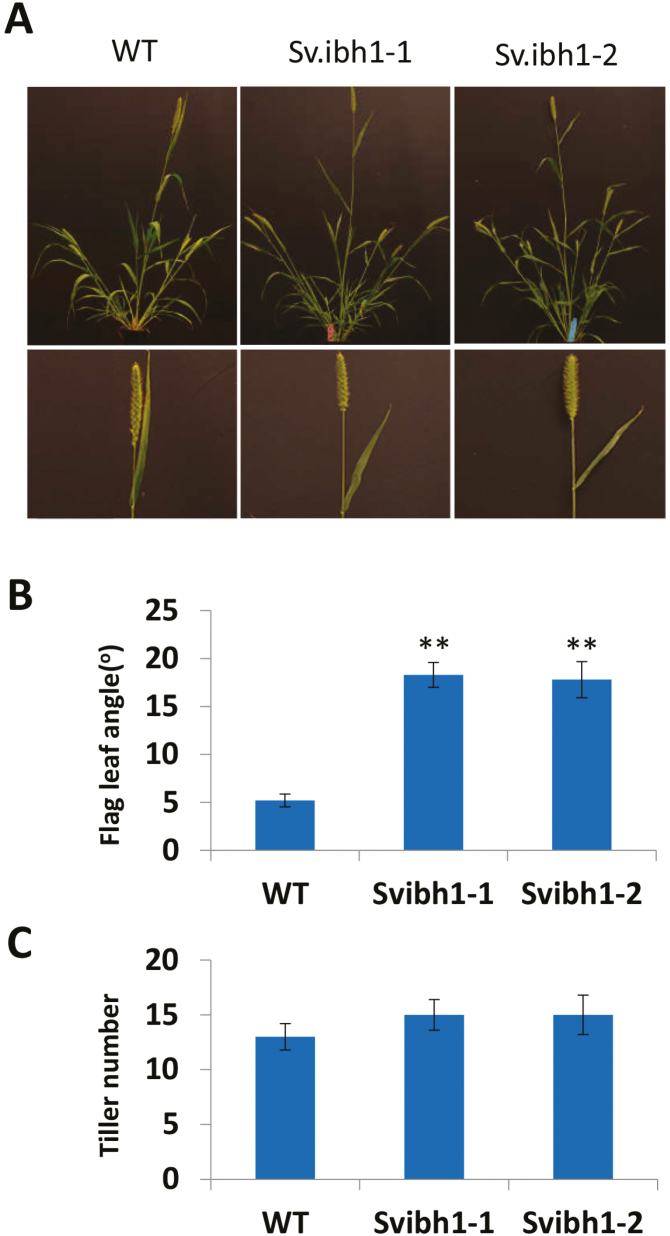
Phenotypic analysis of *Setaria viridis Svibh1-1* mutants. (A) Plant architecture of two mutant lines compared with the wild-type (WT). (B) Flag leaf angle. Data are means (±SD), *n*=100. Significant differences compared with the WT were determined using Student’s *t*-test: **P<0.01. (C) Tiller numbers.

*Setaria viridis* is a panicoid grass that is evolutionarily related to major crops such as maize, rice, and sorghum (Huang *et al.*, 2016). It has a small and simple genome, short stature, short life cycle, produces seeds prolifically, and has simple growing requirements. With the development of genetic and genomic resources over recent years, *Setaria* has been promoted as a model system to address fundamental questions in crop plants. More importantly, *Setaria* has the same leaf architecture as maize, as both are composed of a proximal sheath and a distal blade that are joined together by a hinge-like auricle and membranous ligule. CRISPR-Cas9-mediated genome editing was therefore applied to induce targeted mutagenesis to *SvIBH1-1* to create loss-of-function alleles. Phylogenetic analysis indicated that Sevir3g025700 (*SvIBH1-1*) was the most closely related orthologue to *ZmIBH1-1*(Fig. 1D), and protein sequence alignment showed that it had the same conserved bHLH domain (Fig. S2 at Dryad). *Setaria viridis* ME34 was chosen as the landrace background because it has an erect leaf architecture that makes it easier to screen for altered leaf angle. The knockout mutant had a flag leaf that was bent away from the main panicle rather than wrapping around it (Fig. 2A), the mean LA of which was ~15° (range 10–25°) compared with 5.2° (0–8°) in the wild-type (WT) (Fig. 2B). The mean LA of the next leaf down the stem (second leaf) in *Svibh1-1* was 27.5° (18–36°), compared with 22° in the WT (data not shown). These results indicated that *SvIBH1-1* negatively regulates LA in *Setaria*. No significant differences were found for tiller number (Fig. 2C).

**Fig. 3.**
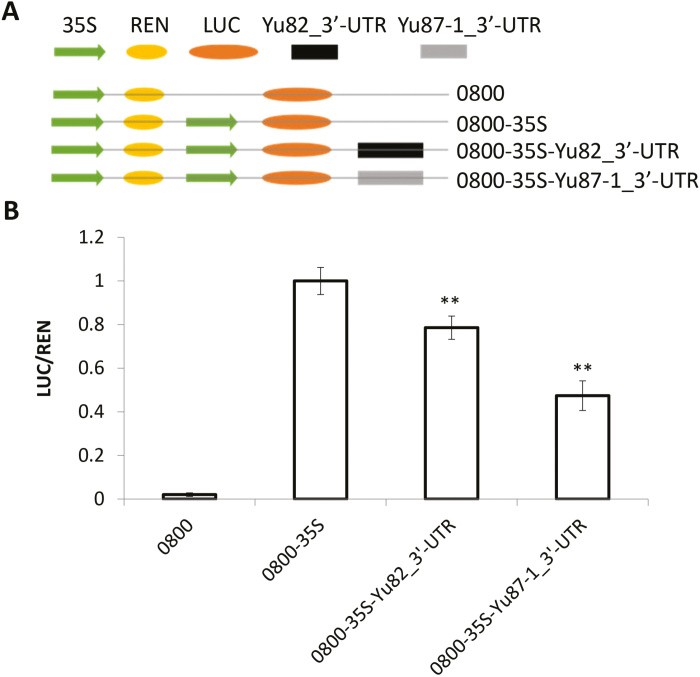
Expression difference test of the 3´-UTRs of *ZmIBH1-1* from the maize Yu82 and Yu87-1 lines in leaves of *Nicotiana benthamiana*. (A) Diagram of the constructs. REN, *Renilla* luciferase; LUC, firefly luciferase; 0800 is the vector. (B) Results of dual-luciferase assays showing the relative expression of LUC/REN. Data are means (±SD). Significant differences compared with the 0800-35S control were determined using Student’s *t*-test: **P<0.01.

Comparison of the genomic sequences of *ZmIBH1-1* in the two parent lines, Yu82 and Yu87-1, showed that there were no variations that would cause amino acid changes in the coding region and no nucleotide changes in the promoter and 5′-UTR. This suggested that variations in the non-coding sequences from the 3´-UTR may underlie the variation in the expression level and/or functionality of *ZmIBH1-1*. Indeed, the main variations between Yu82 and Yu87-1 were found in the region from 1.8 kb to 338 bp downstream of the translational end of *ZmIBH1-1* (Fig. S1 at Dryad). The 3´-UTR is involved in post‐transcriptional regulation, such as mRNA decay rates and the level of mRNA transcripts (Olivas and Parker, 2000; Yu *et al.*, 2007). To test a possible regulatory role of this region in the control of expression, we conducted dual-luciferase transient assays in *N. benthamiana* leaves. The results showed that the LUC reporter gene driven by the 3´-UTR of Yu82 had significantly higher expression than when it was driven by the 3´-UTR of Yu87-1 (Fig. 3), indicating that variations in the non-coding sequences from 3´-UTR could underlie the variations in the expression level and/or functionality of *ZmIBH1-1*.

### Elongation, number, and lignification of cells contribute to the variation in leaf angle

**Fig. 4.**
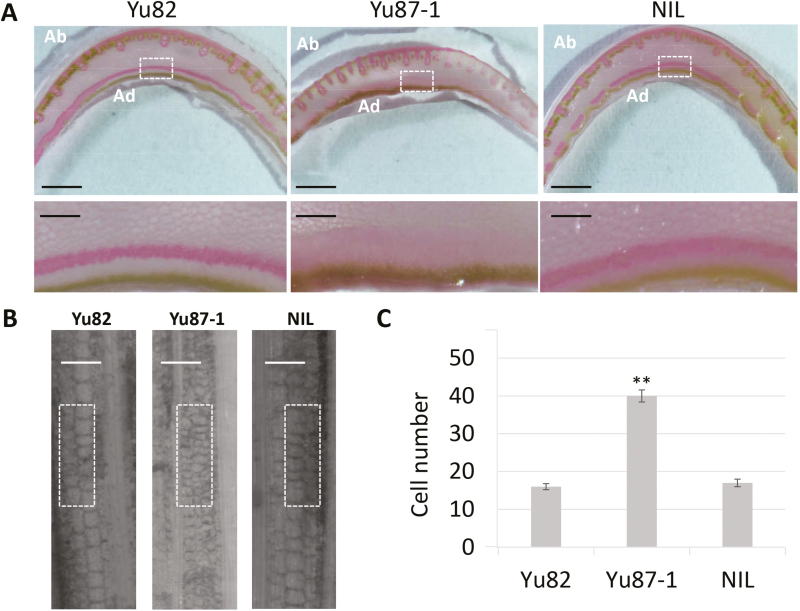
Cytological examination of the parental maize varieties Yu82 and Yu87-1 and the near-isogenic line (NIL) using safranin staining for lignin. The NIL has the same genetic background as Yu87-1 except for the region containing *ZmIBH1-1*, which is derived from Yu82. Sections were taken from fully expanded leaf 7 at the V7 growth stage. (A) Cross-sections of the ligular tissue. The enlarged areas are denoted by the white rectangles. Ab, abaxial; Ad, adaxial. Scale bars are 5 mm in the images above and 0.5 mm in the images below. (B) Longitudinal sections of the ligular tissue. Scale bars are 150 µm. (C) Analysis of cell numbers in the areas denoted by the white rectangles in (B). Data are means (±SD) of *n*=10 replicates. The significant difference between Yu87-1 and the NIL was determined using Student’s *t*-test: **P<0.01.

To investigate cytological changes associated with the decreased LAs in the NILs, safranin staining was performed on fully expanded leaf 7 at the V7 plant growth stage. The NIL and Yu82 had more layers of hypodermal sclerenchyma cells on the adaxial side of the ligular region than Yu87-1 (Fig. 4A), and these cells in the NIL and Yu82 were more lignified than those of Yu87-1. Longitudinal sections of the abaxial surface of the leaf base indicated that cell lengths were smaller and cells were significantly more numerous in Yu87-1 than in the NIL and Yu82 (Fig. 4B, C). These results indicated that the decreased LAs in NILs and Yu82 were caused by more lignified hypodermal sclerenchyma cells in the ligular region, and by increased cell length at the leaf base region.

### RNA-seq identification of genes affected by the differential expression of *ZmIBH1-1*

**Fig. 5.**
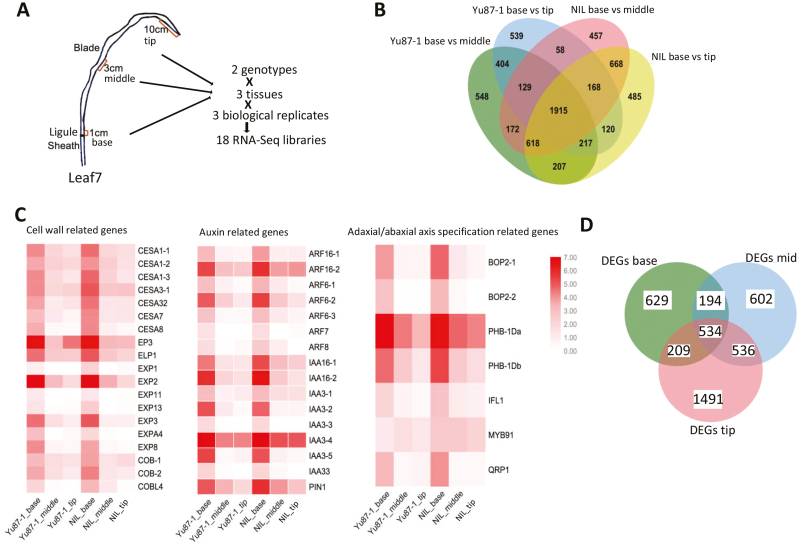
Transcriptomic study of *ZmIBH1-1* of the parental maize variety and Yu87-1 and a near-isogenic line (NIL) having the same genetic background except for the region containing *ZmIBH1-1*, which is derived from Yu82. (A) Summary of the tissues collected and RNA-seq library preparation. (B) Venn diagram showing a comparison of the differentially expressed genes (DEGs) identified in the ligular tissues in Yu87-1 and the NIL. (C) Heat-map show the differences in expression of genes related to the cell wall, auxin, and adaxial/abaxial axis specification. (D) Venn diagram showing a comparison of DEGs in all the tissues between Yu87-1 and NIL.

To understand the regulatory network of *ZmIBH1-1* during the establishment of LA, RNA-seq analysis was conducted using total RNA extracted from different regions of leaf 7 of the Yu87-1 line and the NIL at the V7 growth stage (Fig. 5A). We generated ~8.06 million single-end 100-nt reads for 18 cDNA libraries in total, and 82.51% of the reads uniquely mapped to the B73 reference genome V3 (Table S3 at Dryad).

For quality control, we chose a set of genes related to ligule development to verify tissue specificity. *LG1* is specifically expressed in the ligule region (Johnston *et al.*, 2014), and our results for Yu87-1 and NIL corresponded with this. We found that genes that are known to be preferentially expressed in the ligular region such as *ZmPIN1a*, BOP-*Like*, *BEL14*, and *GA2Ox* (Johnston *et al.*, 2014) also showed significantly high expression levels in the leaf-base tissues of our samples. A total of 214 differentially expressed genes (DEGs) in the ligular tissue (57.3%, out of 373) were detected in our study, which matched the results of Johnston *et al.* (2014) (leaf base; Table S4 at Dryad). Taken together, the results indicated that our data were suitable for further analysis.

The anatomical structures of the ligular region at the base of the leaf blade on both the adaxial and abaxial surface play key roles in leaf development. In order to understand how changes in gene expression regulate development in the ligule region, we first examined the number of genes that were differentially expressed in Yu87-1 and the NIL (Fig. 5B). Genes that showed significant changes in expression (>2-fold, log_2_ fold-change >1) were selected for further analysis, and this resulted in 1915 genes being identified. The genes were functionally annotated using GO enrichment analysis with the online tool agriGO (http://bioinfo.cau.edu.cn/agriGO/). A total of 1347 out of the 1915 genes were functionally annotated, and the most significant subcategory was ‘cell wall organization or biogenesis’ (GO:0071554), which included cellulose synthase genes, such as *ESA*s and *COB*s, and the cell-wall modification genes *EXP*s (Li *et al.*, 2003; Kam *et al.*, 2005) (Fig. 5C). Two other significant subcategories were ‘response to stimulus’ (GO:0050896) and ‘anatomical structure development’ (GO:0048856). Under ‘response to stimulus’, auxin transport and auxin-mediated signaling genes, such as *ARF*s and *IAA*s, were significantly enriched, whilst under ‘anatomical structure development’, genes related to adaxial/abaxial axis specification and to cell growth were significantly enriched (Table S5 at Dryad). A total of 1566 DEGs were identified between Yu87-1 and NIL, 629 of which were specific to the ligular region (leaf base tissue) (Fig. 5D), and 434 (68.9%) of which could be functionally annotated. We conducted GO analysis for these 434 DEGs and determined which ones were related to cellular components. These corresponded to three GO terms, namely ‘membrane’ (GO:0016020, *P*-value=3.9×10^–5^), ‘plant-type cell wall’ (GO:0009505, *P*-value=1.5×10^–5^), and ‘cytoplasmic part’ (GO:0005737, *P*-value=3.5×10^–4^). A total of 132 genes were involved with cellular membranes, 66 of which were specifically associated with the plasma membrane, including *ZmPIP1;6* (*plasma-membrane intrinsic protein1*; also named as *Aquaporin PIP1-6*) and *Bm3* (*brown midrib3*, AC196475.3_FG004). A total of 15 DEGs were related to ‘plant-type cell wall’, including *Expa2* (*alpha expansin2*, GRMZM2G105844), *Expansin A6* (GRMZM2G445169), and *Pectinesterase* (GRMZM2G175499; Arora *et al.*, 2017). A total of 142 genes were related to the cytoplasm, 31 of which were involved in vacuole development (Table S6 at Dryad).

### DAP-seq identification of genes directly targeted by ZmIBH1-1

**Fig. 6.**
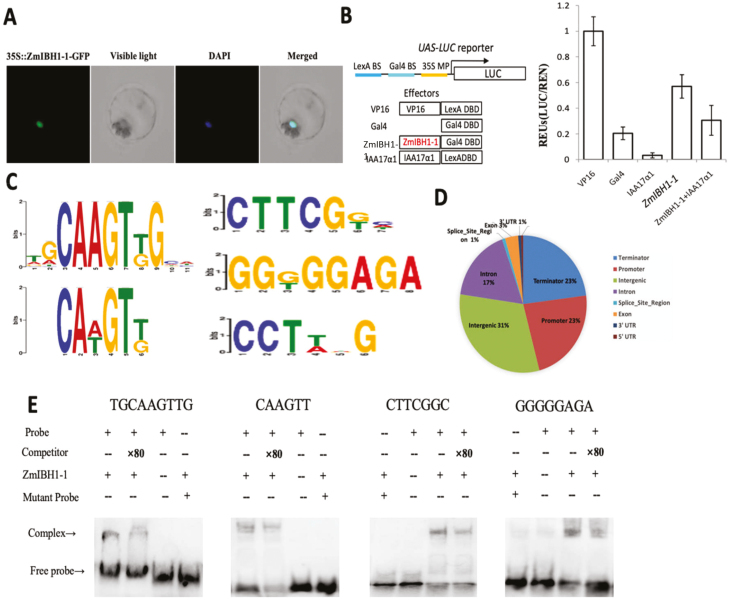
DAP-seq analysis of maize *ZmIBH1-1*. (A) Nuclear localization of ZmIBH1-1 in tobacco protoplasts, as visualized with green fluorescent protein (GFP), with DAPI as the control. (B) Transient assays of transcriptional activity of *ZmIBH1-1*. Protoplasts from a tobacco BY-2 cell line were transformed with the reporter (UAS-LUC) and effector constructs (left), and the reporter gene expression was determined (right). Data are means (±SD), *n*=3. UAS-LUC, reporter construct containing Gal4 and LexA binding sites and a 35S minimal promoter upstream of the coding sequence of LUC; VP16, VP16 fused to the LexA DNA binding domain (DBD); Gal4, Gal4 DBD; IAA17a1, the transcription repression domain of IAA17 fused to the Gal4 DBD; ZmIBH1-1, full-length ZmIBH1-1 fused to Gal4 DBD. The LUC reporter gene expression was normalized to the *Renilla* luciferase activity and presented as values relative to the VP16 control, the value of which was set as 1. (C) ZmIBH1-1 binding to TGCAAGTTGCA, CAAGTT, CTTCGNN, and GGNGGAGA core motifs as identified by MEME-ChIP. (D) Distribution of ZmIBH1-1 binding sites. (E) Results of EMSAs confirming ZmIBH1-1 binding to TGCAAGTTGCA, CAAGTT, CTTCGNN, and GGNGGAGA.

Sequence analysis suggested that *ZmIBH1-1* encodes a bHLH transcription factor, so we first applied the tobacco protoplast system to verify that ZmIBH1-1 is localized to the nucleus (Merkle and Nagy, 1997). The merged images of the GFP and the control DAPI signals indeed suggested that ZmIBH1-1 was localized in the nucleus (Fig. 6A). To confirm that ZmIBH1-1 had the ability to activate or inhibit gene expression, we conducted transcriptional reporter gene assays using a promoter-reporter gene that contained binding sequences for the LexA and Gal4 DNA-binding proteins, The reporter gene was expressed at high levels when co-transformed into protoplasts with the LexA-VP16 fusion construct (Fig. 6B), which contained the coding sequences for the LexA DNA-binding domain (DBD) fused in-frame with the coding sequence of the VP16 transcriptional activation domain. Similar to results reported previously (Tiwari *et al.*, 2001), the fusion LexA DBD with the transcriptional repressor domain of IAA17 (IAA17a1) strongly reduced the activation of the reporter gene. Similarly, co-transformation of the reporter gene with a construct for the ZmIBH1-1-Gal4 fusion protein (ZmIBH1-1-Gal4) significantly increased the expression of the reporter gene (Fig. 6B). These results demonstrated that ZmIBH1-1 specifically induced the expression of *LUC* under the IAA17 repressor domain.

To further explore whether the DEGs identified through RNA-seq analysis were direct targets of ZmIBH1-1, we used DNA affinity purification sequencing (DAP-seq) assays. Using the Illumina platform (150-bp pair-end reads), this produced ~10 million reads in total, 60% of which uniquely mapped to the maize genome V3. ZmIBH1-1 binding sites were examined using MACS2 (Zhang *et al.*, 2008; *q*-value <0.005, based on a Poisson distribution comparing the ZmIBH1-1 sample and the control). Peaks located 5 kb upstream from the transcription start site (TSS) were defined as the peaks located in the promoter region. Peaks located 5kb downstream of the transcription termination site (TTS) were defined as those located in the terminator region. A total of 3189 genes were identified from 4927 peaks identified across the whole genome. Two typical ZmIBH1-1 binding motifs (NNCAAGTNG and CANGTN; Jones, 2004) and two novel binding sites (CTTCGNN and GGNGGAGA) were identified (Fig. 6C). We next analysed the distribution of the peaks within these genes and found that 23% were located within the promoter region (–5 kb to the TSS), 1% were located in the 5´-UTRs, 3% were located in the exon regions, 17% were located in the intron regions, 1% were located in the 3´-UTRs, and 23% were located in the terminator regions (Fig. 6D). To confirm the typical ZmIBH1-1-binding motifs NNCAAGTN and CANGTN, EMSAs were performed using purified ZmIBH1-1 protein and a labelled DNA probe containing the ZmIIBH1-1-binding sites (TGCAAGTTGCA and CAAGTT). As shown in Fig. 6E, ZmIBH1-1 bound to TGCAAGTTGCA and CAAGTT, the addition of 80× unlabeled competitors reduced the detected binding, and it did not bind to mutant probes (TGTGGACTGCA and TAGATAT). Without the ZmIBH1-1 protein only the band for the free probe was observed. The results confirmed the specific binding of ZmIBH1-1 to TGCAAGTTGCA and CAAGTT. In addition, we also found two new ZmIBH1-1-binding motifs, CTTCGNN and GGNGGAGA. An EMSA was performed using a purified ZmIBH1-1 protein and a labelled DNA probe containing the new ZmIIBH1-1-binding sites, CTTCGGC and GGGGAGA. As shown in Fig. 6E, ZmIBH1-1 bound to the two new motifs. The addition of 80× unlabeled competitors reduced the detected binding of ZmIBH1-1, and it did not bind to the mutant probes (ACCTAGC and TTGTTCGA). Without the ZmIBH1-1 protein only the band for the free probe was observed. The results confirmed the specific binding of ZmIBH1-1 to CTTCGGC and GGGGAGA.

**Fig. 7.**
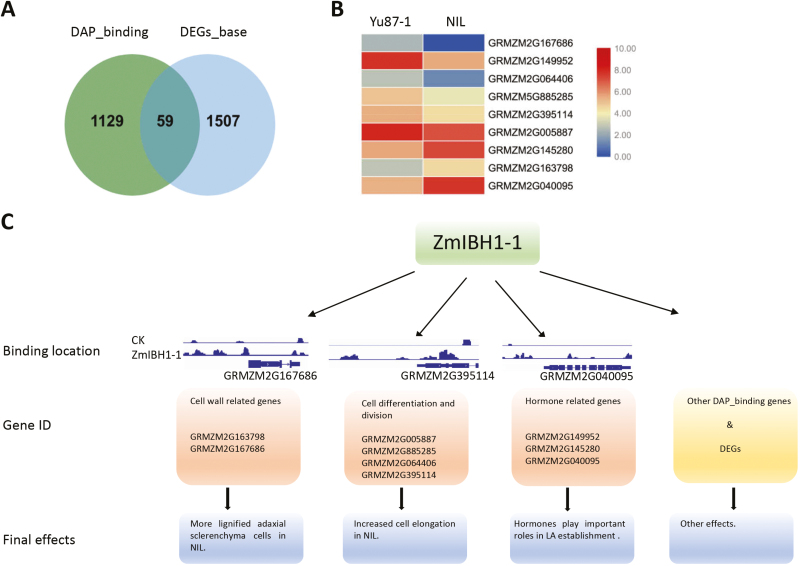
Differentially expressed genes (DEGs) that are bound by ZmIBH1-1, and a proposed ZmIBH1-1 regulatory network. (A) Venn diagram showing a comparison of ZmIBH1-1 binding targets and the DEGs in the ligular region between the parental line Yu87-1 and a near-isogenic line (NIL) having the same genetic background except for the region containing *ZmIBH1-1*, which is derived from Yu82. DAP_binding, ZmIBH1-1 binding targets in the promoter region; DEGs_base, DEGs identified in the ligular region between Yu87-1 and NIL. (B) Heat-map showing the differences in expression of genes related to the cell wall, cell differentiation, and hormones. (C) A hierarchical genome-wide transcriptional regulatory framework for ZmIBH1-1. CK, negative control; LA, leaf angle.

Of the 3189 target genes bound by ZmIBH1-1, only 1188 bound to the upstream regions. RNA-seq analysis revealed 1566 DEGs between the leaf base of Yu87-1 and the NIL, and DAP-seq identified 59 of these as putative target genes where ZmIBH1-1 bound to upstream regions (Fig. 7A). Of these 59 genes, 29 were up-regulated in Yu87-1 and 30 were down-regulated, and genes related to the cell wall, , cell development, and hormone metabolism were identified as being the major components (Fig. 7B; Table S7 at Dryad).

### ZmIBH1-1 directly regulates genes involved in cell wall metabolism

By aligning the target genes of ZmIBH1-1 to the DEGs, two genes were identified as related to cell wall biosynthesis or modification (Fig. 7C). *Walls Are Thin1* (*WAT1*, GRMZM2G163798) was significantly up-regulated in the NIL and corresponded to more lignified adaxial sclerenchyma cells and more elongated cells in the NIL (Fig. 4). *AtWAT1* is a gene required for secondary wall formation in Arabidopsis, and the *wat1* mutant shows defects in stem-fiber cell wall thickness and cell elongation, and has reduced lignin and stem strength (Ranocha *et al.*, 2011). In addition to *WAT1*, a gene related cell wall modification, GRMZM2G167686, was expressed in Yu87-1 but not in the NIL. To determine whether ZmIBH1-1 directly regulated these genes we performed dual-luciferase transient transcriptional activity assays in *N. benthamiana* leaves with ZmIBH1-1driven by the CaMV 35S promoter as the effecter and LUC as the reporter gene (Fig. S3A at Dryad). The results showed that ZmIBH1-1 specifically increased the expression of LUC with the *WAT1* promoter and it repressed the promoter of GRMZM2G167686, indicating these genes are indeed targets of ZmIBH1-1. The results were consistent with the corresponding gene expression results.

### ZmIBH1-1 directly regulates genes related to cell proliferation

Four genes related to cell development were targeted directly by ZmIBH1-1, and all of them were up-regulated in Yu87-1 compared to the NIL (Table S7 at Dryad). GRMZM2G005887 and GRMZM5G885285 were annotated as a proteins related to cell differentiation, and GRMZM2G064406 and GRMZM2G395114 were annotated as proteins related to cell division. We performed dual-luciferase transient transcriptional activity assays with the ZmIBH1-1 protein and LUC driven by the promoter sequences of the four genes as reporters in *N. benthamiana* (Fig. S3A at Dryad). The results showed that the promoters of all the genes were significantly induced by ZmIBH1-1, indicating the genes are targets of ZmIBH1-1 (Fig. S3B at Dryad). These data confirmed the important roles of genes related to cell differentiation and proliferation in the regulation of leaf architecture.

### ZmIBH1-1 is involved in hormone regulation

Plant hormones play pivotal roles in the formation of LA (Luo *et al.*, 2016). Previous studies have shown that cytokinins and jasmonates affect the inclination of the lamina by interacting with other phytohormones, such as auxins and brassinosteroids (Eizo, 1965; Wada *et al.*, 1984; Gan *et al.*, 2015). In our analysis of the transcriptome in the leaf ligular region, genes involved in cytokinin, jasmonate, and ethylene synthesis and signal transduction pathways were detected (Table S7 at Dryad). The genes GRMZM2G145280 and GRMZM2G149952 were both responsive to cytokinin, but GRMZM2G149952 was down-regulated in the mutant NIL whilst GRMZM2G145280 was up-regulated. A jasmonate biosynthesis gene, GRMZM2G040095, was detected and had 16-fold higher abundance of transcript accumulation in the NIL than in Yu87-1.

## Discussion

### Map-based cloning of *ZmIBH1-1*

Using a nested-association mapping population, Tian *et al.* (2011) detected three major QTLs related to leaf angle (LA) on chromosome 2, and *qLA2-1* was located in one of these. Cloning the candidate gene within this region may therefore contribute to the understanding of the molecular mechanisms controlling LA in maize.

Our study was based on the development of 300 new SSR markers and 60 polymorphic primers were identified from the screening of the parental lines (Table S2 at Dryad). The large fine-mapping populations that were used in this study increased the recombination rate of different chromosome segments, and thus improved the accuracy of the mapping results. In addition, we combined the phenotype with the genotype in the process of constructing the NIL population, and a single plant with a background recovery rate >95% with LA <15° was selected (Ku *et al.*, 2010). As well as increasing the size of the fine-mapping population, we also increased the mapping efficiency and accuracy. Map-based cloning has not only been widely used in the isolation of small-genome crop species such as rice, it has also become an important method for gene isolation of large-genome crops, such as maize, and this will benefit crop breeding projects.

### bHLH transcription factors play important roles in regulating LA in maize

In plants, bHLH family proteins such as MYCs, BEEs, PRE (PACLOBUTRAZOL RESISTANCE), BIM (BES1-INTERACTING MYC-LIKE), and PIFs (PHYTOCHROME INTERACTING FACTORS) have been shown to function in multiple cellular processes, including plant morphogenesis, epidermal cell differentiation, shade-avoidance responses, and stress responses (Ledent and Vervoort, 2001; Heim, 2003; Duek and Fankhauser, 2005). The maize genome encodes a total of 262 bHLH proteins grouped into 21 subfamilies (Feller *et al.*, 2011). bHLH transcription factors are a group of proteins containing 60 amino acids with two distinctive regions, the basic region and the HLH region. The former consists of 13–17 highly conserved residues (His, Glu, Arg) and is arranged at the domain N-terminal end, and mediates the recognition process between the bHLH monomer and the DNA–protein binding site. The HLH region carries two amphipathic alpha-helices linked by a variable loop, which allows them to bind as dimers (Atchley and Fitch, 1997). Previous studies have indicated that bHLH proteins are involved in a wide range of plant developmental and physiological activities, including plant morphogenesis, stomatal differentiation, and cell elongation (Nadeau, 2009; Zhang *et al.*, 2009; Ikeda *et al.*, 2013). There are no reports that bHLH functions in the regulation of maize leaf architecture. It has been reported that all six members of the bHLH subfamily in rice have a conserved function in regulating the LA of the flag leaf (Dong *et al.*, 2018). In maize, the first bHLH family member identified was the *R* gene, which is essential for anthocyanin synthesis (Ludwig and Wessler, 1990); however, only a few bHLH proteins have been functionally characterized so far in maize. Here, we cloned ZmIBH1-1, encoding a bHLH transcription factor through map-based cloning. Cytological examination revealed an increased number of sclerenchymatous cells in the leaf base area of the NIL (Fig. 4). The abnormal mechanical tissues in the NIL leaf-base area were also more lignified, which would lead to stronger mechanical support for the leaves. Thus, ZmIBH1-1 regulated LA through affecting the number of sclerenchymatous cells and producing abnormal mechanical tissues. In addition, a mutant of *Setaria* generated using CRISPR-Cas9 technology to knockdown *SevirIBH1* showed increased LA (Fig. 2), confirming a negative role of *IBH1* in leaf angle and thus implying that *ZmIBH1-1* negatively regulates LA in maize. In rice, OsIBH1 also acts as a negative regulator and plays an important role in the regulation of LA (Zhang *et al.*, 2009).

### ZmIBH1-1 directly regulates specific genes

Histological analysis combined with RNA-seq and DAP-seq results indicated that ZmIBH1-1 regulated cell wall lignification, proliferation, and elongation within the ligular region of the maize leaf to affect the LA (Figs 4–6). ZmIBH1-1 directly bound to *WAT1* (Fig. 7), which encodes a plant-specific protein that dictates secondary cell wall thickness of fibers in Arabidopsis (Ranocha *et al.*, 2011). The *wat1* mutant leads to decreased fiber cell wall thickness in Arabidopsis stems. We found that the expression level *WAT1* was significantly higher in the NIL than in Yu87-1 and that cell wall lignification was also higher in the NIL. Taken together, this suggests that fiber cell wall thickness is a main factor determining LA. Jasmonate acts in plant growth and development (Huang *et al.*, 2017), and bHLH transcription factors in Arabidopsis including MYC2 and its homologs MYC3–5 all interact with JAZ proteins (Cheng *et al.*, 2011; Fernández-Calvo *et al.*, 2011; Niu *et al.*, 2011; Qi *et al.*, 2015). The jasmonate synthesis/degradation gene GRMZM2G040095 had higher abundance of transcripts in the NIL than in Yu87-1 (Fig. 7). In addition, cytokinin has been reported to regulate leaf axil establishment in maize (Wang *et al.*, 2014*a*), and GRMZM2G145280 (*BBC1*) and GRMZM2G149952 were both annotated as ‘response to cytokinin’ (Table S7 at Dryad). *BBC1* activity is positively correlated with active cell division in Arabidopsis (Bertauche *et al.*, 1994). The expression level of *BBC1* was higher in NIL than in Yu87-1, which implies that it also affected the formation of LA through cell division.

## Conclusions

We have used map-based cloning to identify *ZmIBH1-1* as a negative regulator of leaf angle (LA) in maize. Histological analysis showed that differences in LA between two parental lines and a NIL were mainly the result of differential cell wall lignification and cell elongation in the ligular region. RNA-seq and DAP-seq analyses identified 59 target genes of ZmIBH1-1, with annotation that were related to either the cell wall, cell development, or hormones. Our results from GO enrichment analysis and cytological studies indicated that ZmIBH1-1 affected LA mainly through regulation of cell wall lignification and cell elongation. We present a model of gene regulation by ZmIBH1-1 to explain the control of maize LA (Fig. 7C).

Our results demonstrate that *ZmIBH1-1* encodes a transcription activator of multiple metabolic pathways related to cell metabolism during the establishment of LA in maize, and it acts in both direct and indirect ways to affect the patterns of gene transcription. Our data suggest that ZmIBH1-1 not only directly activates genes associated with a variety of genes related to cell development, but also controls transcription factors that, in turn, regulate various other aspects of plant metabolism. A better understanding of the regulatory pathways of *ZmIBH1-1* will provide insights into the pleiotropic effects of the bHLH transcription factors on the establishment of plant architecture in maize, and will help to support future efforts to breed high-yielding varieties.

## Data availability

The following data are available at Dryad Data Repository: http://dx.doi.org/10.5061/dryad.18tk64p.

Fig. S1. Sequence alignment of Yu82 and Yu87-1.

Fig. S2. Protein sequence alignment of ZmIBH1-1 and SvIBH1-1.

Fig. S3. Results of dual-luciferase transient transcriptional activity assays

Table S1. Summary of the primary mapping of *qla2-1*.

Table S2. Primers used in this study.

Table S3. Summary of RNA-seq mapping.

Table S4. Verification of RNA-seq results.

Table S5. AgriGo analysis of genes differentially expressed in the ligular region of both NIL and Yu87-1.

Table S6. AgriGo analysis of genes differentially expressed in the ligular region in either NIL and Yu87-1.

Table S7. Differentially expressed genes detected in the DAP-seq analysis.

eraa052_suppl_Supplementary_Figures

eraa052_suppl_Supplementary_Table_S1

eraa052_suppl_Supplementary_Table_S2

eraa052_suppl_Supplementary_Table_S3

eraa052_suppl_Supplementary_Table_S4

eraa052_suppl_Supplementary_Table_S5

eraa052_suppl_Supplementary_Table_S6

eraa052_suppl_Supplementary_Table_S7
