# Supplementary material for: ZmIBH1-1 regulates plant architecture in maize
Source: J Exp Bot. 2020 Jan 28;71(10):2943–55. doi: 10.1093/jxb/eraa052 (PMC7260713; doi:10.1093/jxb/eraa052)
Supplement: eraa052_suppl_Supplementary_Figures [file eraa052_suppl_supplementary_figures.pdf]

```
Yu82.seq      ATGGCCAGGAAGAGGACGGCGGCGCGCCATCAGCACCAGGAACCACCGCCAAACCCTAACCCTAACCTCCGCCGCCGGGCCGCGGCCAGCGACCCGGCCT   100
Yu87-1.seq    ATGGCCAGGAAGAGGACGGCGGCGCGCCATCAGCACCAGGAACCACCGCCAAACCCTAACCCTAACCTCCGCCGCCGGGCCGCGGCCAGCGACCCGGCCT   100
Consensus     atggccaggaagaggacggcggcgcgccatcagcaccaggaaccaccgccaaaccctaaccctaacctccgccgccgggccgcggccagcgacccggcct

Yu82.seq      CGGACGAGCCGCCGCCGTCGTCGAAGCGCATGCTGGCCTTTCACTTCCTCCGCGCGCTGGCCAGGATCCACAGCACCACCCCGGCGCCGCGCCGCCCGCG   200
Yu87-1.seq    CGGACGAGCCGCCGCCGTCGTCGAAGCGCATGCTGGCCTTTCACTTCCTCCGCGCGCTGGCCAGGATCCACAGCACCACCCCGGCGCCGCGCCGCCCGCG   200
Consensus     cggacgagccgccgccgtcgtcgaagcgcatgctggcctttcacttcctccgcgcgctggccaggatccacagcaccaccccggcgccgcgccgcccgcg

Yu82.seq      CACCATCCGCCGCGCGGCCTACTCGTCCATGGCGCGCGCTGCCAGCCCACGCCGGGCCTGGACGCAGGCGCTGCTCCGCCAGGCTCGCGCGCGCAGGGCG   300
Yu87-1.seq    CACCATCCGCCGCGCGGCCTACTCGTCCATGGCGCGCGCTGCCAGCCCACGCCGGGCCTGGACGCAGGCGCTGCTCCGCCAGGCTCGCGCGCGCAGGGCG   300
Consensus     caccatccgccgcgcggcctactcgtccatggcgcgcgctgccagcccacgccgggcctggacgcaggcgctgctccgccaggctcgcgcgcgcagggcg

Yu82.seq      GCTGCCAGGTCCTCCAGGGGAGCCGTCCTGCTGCGGAGACGCGTCGCCTCCGCCGCGGCGTCTCCTCCGCCGCCGCTGCTCCGCGCCAGCGCCGGGGAGT   400
Yu87-1.seq    GCTGCCAGGTCCTCCAGGGGAGCCGTCCTGCTGCGGAGACGCGTCGCCTCCGCCGCGGCGTCTCCTCCGCCGCCGCTGCTCCGCGCCAGCGCCGGGGAGT   400
Consensus     gctgccaggtcctccaggggagccgtcctgctgcggagacgcgtcgcctccgccgcggcgtctcctccgccgccgctgctccgcgccagcgccggggagt

Yu82.seq      CGACGTCGGCGCCGACGCCGCTGG...CTCCGGCGGCCGTGGCGGCCCGGGGCCCGCCTCCGAGGCAGGCCGGGGAACCGGCCAGGGCCGACGCGCTCCG   497
Yu87-1.seq    CGACGTCGGCGCCGACGCCGCTGGTGGCTCCGGCGGCCGTGGCGGCCCGGGGCCCGCCTCCGAGGCAGGCCGGGGAACCGGCCAGGGCCGACGCGCTCCG   500
Consensus     cgacgtcggcgccgacgccgctggtggctccggcggccgtggcggcccggggcccgcctccgaggcaggccggggaaccggccagggccgacgcgctccg

Yu82.seq      CCGGCTCGTCCCCGGCGGCGCGGAGATGGAGTACGGCAGCCTCCTCGACGAGACCGCCGACTACGTGCGCTCCCTCCGCGCGCAGGTGCAGCTCATGCAG   597
Yu87-1.seq    CCGGCTCGTCCCCGGCGGCGCGGAGATGGAGTACGGCAGCCTCCTCGACGAGACCGCCGACTACGTGCGCTCCCTCCGCGCGCAGGTGCAGCTCATGCAG   600
Consensus     ccggctcgtccccggcggcgcggagatggagtacggcagcctcctcgacgagaccgccgactacgtgcgctccctccgcgcgcaggtgcagctcatgcag

Yu82.seq      AGCCTCGTCGACCTCTTCTCCGCCCAATGATCGATCGTCCAACAACCGAACCAGTAATTAATTAATACCGCATGGTTAATTAATTCTTGTTCTAGCTTGC   697
Yu87-1.seq    AGCCTCGTCGACCTCTTCTCCGCCCAATGATCGATCGTCCAACAACCGAACCAGTAATTAATTAATACCGCATGGTTAATTAATTCCTGTTCT....TGC   696
Consensus     agcctcgtcgacctcttctccgcccaatgatcgatcgtccaacaaccgaaccagtaattaattaataccgcatggttaattaattcctgttctagcttgc

Yu82.seq      ACCGTCCATCGTTCGCCGCGCCGCCGCCTTTCGTATCTATATCATCGCATATCTCTTCTTCGTGCCACATATGAGATGGATGAGTGATTAAGATAGGAGA   797
Yu87-1.seq    ACCGTCCATCGTTCGCCGCGCCGCCGCCTTTCGTATCTATATCATCGCATATCTCTTCTTCGTGCCACATATGAGATGGATGAGTGATTAAGATAGGAGA   796
Consensus     accgtccatcgttcgccgcgccgccgcctttcgtatctatatcatcgcatatctcttcttcgtgccacatatgagatggatgagtgattaagataggaga

Yu82.seq      TTATTGATAAGAGATTGTTATTGCTGGTACATATATATGTTTAGTTGGTAAGATAAGATTATTAGTAGGTGTATGTATAGGTTGGGAACATACATGCATG   897
Yu87-1.seq    TTATTGAGAAGAGATTGTTATTGCTGGTACATATATATATTTAGCTGGTAAGATAAGATTATTAGTAGGTGTATGTATAGGTTGGGAACATACATGCATG   896
Consensus     ttattgagaagagattgttattgctggtacatatatatatttagctggtaagataagattattagtaggtgtatgtataggttgggaacatacatgcatg

Yu82.seq      GAAGATGGTACCACGCACACACACCAAGCAGCAATTGGAGGGAGAGGAGATTATTATTGAGGTAATTAATTGTAACATATATATAGAGAGAATTGAAGCA   997
Yu87-1.seq    GAAGATGGTACCA....CACACACCAAGCAGCAATTGGAGGGAGAGGAGATTATTATTGAGGTAATTAATTGTAACATATATAGAGAGAGAATTGAAGCA   992
Consensus     gaagatggtaccacgcacacacaccaagcagcaattggagggagaggagattattattgaggtaattaattgtaacatatatagagagagaattgaagca

Yu82.seq      TGGAGTCACATACATACATACATAAGCTATTAGTGCAGTGCTTGCTGTTGGGTCAGATGATTGGGTATGCATATATGTTTGTAAAAAATTGTGGAGCCAT   1097
Yu87-1.seq    TGGAGCCACATACATACATACATAAGCTATTAGTGCAGTGCTTGCTGTTGGGTCAGATGATTGGGTATGCATATATATTTGTAAAAAATTGTGGAGCCAT   1092
Consensus     tggagccacatacatacatacataagctattagtgcagtgcttgctgttgggtcagatgattgggtatgcatatatatttgtaaaaaattgtggagccat

Yu82.seq      GTCATG                                                                                                1103
Yu87-1.seq    GTCATG                                                                                                1098
Consensus     gtcatg
```

Supplementary figure1. Sequence alignment of Yu82 and Yu87-1. Polymorphisms are highlighted in cyan and the predicted translation start of ZmIBH1-1 is boxed in yellow and the predicted termination codon of ZmIBH1-1 is boxed in red.

```
GRMZM2G388823      MARKRTAARHQHQEPPPNPNPNLRRRAAASDPASDEPPPS-----SKRMLAFHFLRALAR
Sevir.3G025700     MARKRTAR--PPPPPPPNPNPNPNRRAVASSAATAPDPASGSASPSKRMLAFHFLRALAR
                   *******       ******** .***.**..*:   *.*     ***************

GRMZM2G388823      IHSTTPAPRRPRTIRRAAYSSMARAASPRRAWTQALLRQARARRAAARSSRGAVLLRRRV
Sevir.3G025700     IHSATPVPRRTRNIRRAAYSSMARAANPRRAWTQALLRQVRVCRAMRSRRAVLLRRRVSG
                   ***:**.***.*.*************.************.*. **        :  *

GRMZM2G388823      ASAAASPPPPLLRASAGESTSAPTPLAPAAVAARGPPPRQAGEPARADALRRLVPGGAEM
Sevir.3G025700     APPAASPAPPLGAARSTVSAAGGTSAAAAALPRGGPPPRQAGEPARADALRRLVPGGSEM
                   *..****.***  * :  *::. *. *.**:.  ***********************:**

GRMZM2G388823      EYGSLLDETADYVRSLRAQVQLMQSLVDLFSAQ
Sevir.3G025700     EYCSLLDETADYLRCLRAQVQLMQNLVDLFSGQ
                   ** *********:*.*********.******.*
```

Supplementary figure 2. Protein sequence alignment of ZmIBH1-1 and SvIBH1-1. Red squares indicate the conserved bHLH domain. '*' indicates positions which have a single, fully conserved residue. ':' indicates that one of the following 'strong' groups is fully conserved: STA, NEQK, NHQK, NDEQ, QHRK, MILV, MILF, HY, and FYW. '.' indicates that one of the following 'weaker' groups is fully conserved: CSA, ATV, SAG, STNK, STPA, SGND, SNDEQK, NDEQHK, NEQHRK, FVLIM, and HFY.

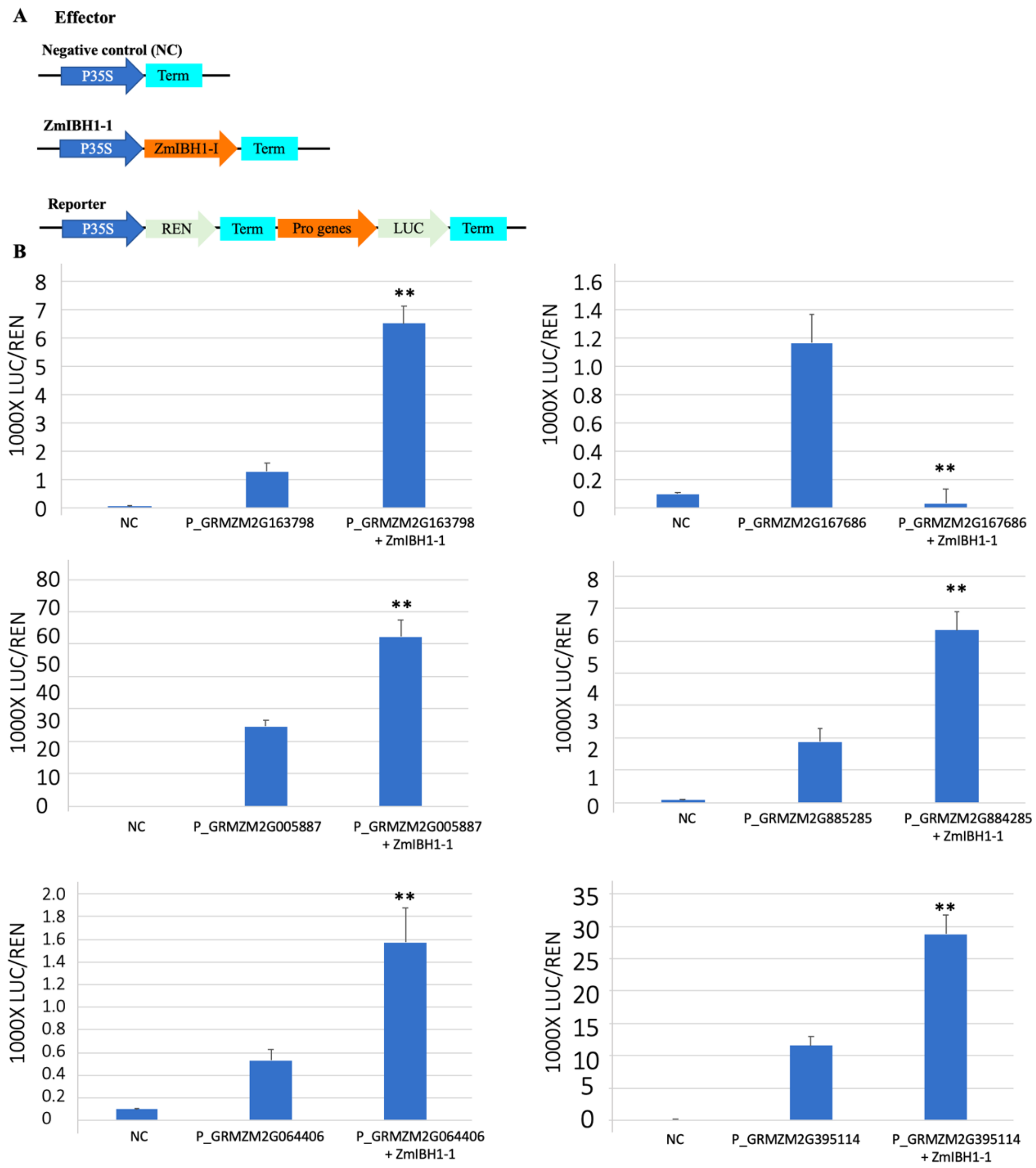

Supplementary figure 3. Results of the dual-luciferase transient transcriptional activity assay. (A) The 35S:REN-Pro zein:LUC reporter constructs were transiently expressed in onion epidermal cells together with control vector or 35S:ZmIBH1-1effector, respectively. (B) The expression level of Renilla (REN) was used as an internal control. The LUC/REN ratio represents the relative activity of candidate gene promoters. Data are values of three independent experiments. Significant differences from the corresponding control values (using Student's t test [n = 3]): *P < 0.05, **P < 0.01, and ***P < 0.001.
